# Nursing Exposure to Bisphenols as a Cause of Male Idiopathic Infertility

**DOI:** 10.3389/fphys.2022.725442

**Published:** 2022-02-24

**Authors:** Tereza Fenclová, Hedvika Řimnáčová, Marouane Chemek, Jiřina Havránková, Pavel Klein, Milena Králíčková, Jan Nevoral

**Affiliations:** ^1^Biomedical Center, Faculty of Medicine in Pilsen, Charles University, Pilsen, Czechia; ^2^Department of Histology and Embryology, Faculty of Medicine in Pilsen, Charles University, Prague, Czechia

**Keywords:** bisphenol, DNA damage, idiopathic infertility, spermatogenesis, nursing exposure

## Abstract

Idiopathic infertility is a serious problem, which can be caused and explained by exposure to endocrine disruptors, such as bisphenols. In our study, we studied transactional exposure to bisphenol and its effects on newborn male mice throughout their reproductive life. Newborn male mice were exposed to bisphenol S and bisphenol F through maternal milk from post-natal day 0 to post-natal day 15 at concentrations of 0.1 ng.g/bw/day and 10 ng.g/bw/day, respectively. Although there were minimal differences between the control and experimental groups in testicular tissue quality and spermatozoa quality, we discovered an interesting influence on early embryonic development. Moderate doses of bisphenol negatively affected cleavage of the early embryo and subsequently, the blastocyst rate, as well as the number of blastomeres per blastocyst. In our study, we focused on correlations between particular stages from spermatogenesis to blastocyst development. We followed epigenetic changes such as dimethylation of histone H3 and phosphorylation of histone H2 from germ cells to blastocysts; we discovered the transfer of DNA double-strand breaks through the paternal pronucleus from spermatozoa to blastomeres in the blastocyst. We elucidated the impact of sperm DNA damage on early embryonic development, and our results indicate that idiopathic infertility in adulthood may have causes related to the perinatal period.

## Introduction

The impact of the environment on reproductive health may be an explanation for idiopathic infertility (Shi et al., 2017). Environmental contaminants with estrogenic and/or anti-androgenic activity include bisphenols (BPs) and endocrine disruptors (Glausiusz, 2014; Eladak et al., 2015; Rahman et al., 2015). Bisphenols are mostly present in manufactured plastics, paper, cans, and other products for daily use (Simoneau et al., 2011; Wong and Durrani, 2017). The most widely used bisphenol, bisphenol A (BPA), has various deleterious effects on human physiology and health, including male (Salian et al., 2011; Rahman et al., 2015; Shi et al., 2019) and female (Rivera et al., 2015) reproduction and fertility (Vandenberg et al., 2007; Siracusa et al., 2018) as well as *in vitro* fertilization (IVF) (Mok-Lin et al., 2010). This effect has been observed even at very low (e.g., subtoxic) concentrations (Vandenberg et al., 2012; Eladak et al., 2015). BPA has been replaced in the manufacturing process with several analogs, including bisphenol S (BPS) and bisphenol F (BPF) (Eladak et al., 2015; Shi et al., 2019), which are more chemically stable than BPA but are worse in terms of biodegradability; they also show better dermal penetration (Ike et al., 2006; Danzl et al., 2009; Liao et al., 2012a,b). Bisphenol A analogs contaminate the environment (Chen et al., 2016), including water, air, house dust, and food (Ike et al., 2006; Viñas et al., 2010; Liao and Kannan, 2014; Wu et al., 2018). Therefore, the highest intake of bisphenols in humans occurs through the diet, primarily through the consumption of canned foods or drinking bottled water (Ehrlich et al., 2014; Eladak et al., 2015). However, we can consider more inconspicuous routes of exposure, such as gestational and/or perinatal exposure via the placenta and breast milk, respectively.

Prenatal exposure to bisphenols in male rats decreased sperm motility, counts, and quality (Salian et al., 2011). In addition, changes in testicular peripubertal development (Muñoz-de-Toro et al., 2005), testicular tissue, and testes gene expression have been observed in mice (Shi et al., 2018), and these changes were proven to be transferred to the next generation (Salian et al., 2011; Rahman et al., 2017). Moreover, bisphenols affect post-translational modifications in sperm, and dimethylation of lysine K4 on histone H3 (H3K4me2) has been established as an indicator of aberrant histone-protamine exchange, resulting in improper chromatin condensation (Štiavnická et al., 2020) and chromatin immaturity (Lambrot et al., 2019; Štiavnická et al., 2020). The epigenetic mode of action is similarly affected by bisphenol in oocytes (Žalmanová et al., 2017; Nevoral et al., 2018; Prokešová et al., 2020).

However, perinatal nursing exposure, as another indirect exposition route is currently underestimated, although the impact seems to be significant for the following reasons: (i) bisphenols are more concentrated in maternal milk; (ii) maternal milk (with bisphenols) is an exclusive food for newborns; and (iii) newborns do not have fully developed detoxification mechanisms, especially in the liver and kidneys (Matalová et al., 2016). In our experimental approach, dams were exposed to bisphenols via a common route of exposure during the breastfeeding period, leading to indirect exposure of their offspring (Chemek and Nevoral, 2019). The dosage of BPs is considered to be significant (EFSA Panel on Food Contact Materials, Enzymes, and Flavourings and Processing Aids [CEF], 2015).

In our experiment, we studied transactional bisphenol exposure and its effects on newborn male mice throughout their reproductive life. What is currently diagnosed as idiopathic infertility can, in fact, be the result of exposure to endocrine disruptors during the nursing period. Our study is unique owing to very low bisphenol doses in the early exposure window with consequences in adulthood, which is a model of idiopathic infertility. Our innovative approach revealed correlations between each stage of spermatogenesis, with overlap to early embryonic development, zygotes, and blastocysts.

## Materials and Methods

### Chemicals

All basic chemicals were purchased from Sigma-Aldrich (Missouri, United States), and chemicals for sperm washing were purchased from Irvine Scientific (California, United States) unless otherwise noted. Antibodies against anti-H3K4me2 and anti-γH2AX and anti-mouse/rabbit IgG antibodies conjugated with fluorescein (Alexa Fluor 488/647) were purchased from Abcam (Cambridge, United Kingdom). Anti-tubulin antibody was used as an internal loading control and was purchased from Cell Signaling Technology (Massachusetts, United States). Anti-mouse and anti-rabbit antibodies conjugated with horseradish peroxidase were purchased from Sigma-Aldrich (Missouri, United States).

All animal procedures were conducted in accordance with Act No. 246/1992 Coll. on the Protection of Animals Against Cruelty under the supervision of the Animal Welfare Committee of the Ministry of Education, Youth and Sports of the Czech Republic, approval ID MSMT-11925/2016-3.

### Animals

Six- to seven-week-old female ICR mice were purchased from Velaz Ltd. (Czech Republic) and used as mothers of F1 offspring subjected to experimental assessment. Experimental design consisted of six groups; vehicle control, low diethylstilbestrol (DES), low BPS, moderate BPS, low BPF, and moderate BPF. We exposed nursing dams (at least *n* = 6 in each experimental group) of outbred ICR mice to low and moderate levels of BPS and BPF (0.2 and 20 ng/g body weight of dams per day, respectively) over the first 15 days of nursing. Within 18 months, we analyzed a total of 82 of their litters, when each group consisted of at least seven litters. We analyzed at least three male pups from each litter. All animals were housed in intact polysulfonate cages and maintained in a facility with a 12-h light/dark cycle, a temperature of 21 ± 1°C, and relative humidity of 60%. A phyto-estrogen-free diet (1814P; Altromin, Germany) and ultrapure water (in glass bottles, changed twice per week) were provided *ad libitum*.

### Bisphenol Dosing

Dams were dosed with BPS or BPF through drinking water, 0.375 ng/mL and 37.5 ng/mL (hereafter denoted as low and moderate concentrations, respectively), and the other animals were exposed to the vehicle (0.1% ethanol). Dams were treated from delivery until the 15th post-natal day (PND) of the male offspring. Doses were chosen in accordance with known biological effect published previously (Nevoral et al., 2018; Prokešová et al., 2020) and to obtain an assumed exposure to 0.1 ng.g/body weight/day BPS and 10 ng.g/body weight/day BPF. The route of the exposure was used with respect to the welfare of nursing dams and real intake was precisely calculated within genuine water intake as 0.2 ng/g body weight/day and 20 ng/g body weight/day for BPS and BPF, respectively. The male offspring were weaned at PND 21 and housed individually in standard conditions until the 14th week of age, when reproductive maturity was achieved.

### Embryo Flushing and *in vitro* Culture

Eight-week-old embryo donors were stimulated with pregnant mare serum gonadotropin and human chorionic gonadotropin 48 h later. Following overnight mating, females were euthanized by cervical dislocation, and one-cell zygotes were isolated from the oviduct in 0.1% hyaluronidase in M2 medium. After cumulus cell removal, zygotes were fixed in 4% paraformaldehyde for 30 min and stored at 4°C until further experimentation. Alternatively, zygotes were cultured for an additional 4 days *in vitro*, using the modified potassium simplex optimization medium covered with mineral oil. At the end of embryo culture, the blastocyst rate was recorded, and blastocysts were fixed as mentioned above and used for further experiments. The experimental males were euthanized by cervical dislocation. The cauda epididymis was isolated into human tubal fluid medium with 0.1% bovine serum albumin. Sperm were allowed to swim for 30 min and used for further experiments.

### Sperm Characteristics and Assessment

Sperm concentration and motility were evaluated using a prewarmed Makler chamber and a light microscope (Olympus CKX 41; Germany) equipped with a 10 × objective lens (CAchN NA 0.25). Ten microliters of sperm suspension were placed into the Makler chamber. Thereafter, the average sperm concentration (million per milliliter) was counted in three lines, each of 10 squares, and divided by three. Simultaneously, each spermatozoon across the counted area was identified as either motile or immotile. Motility was expressed as the percentage of motile sperm from the total count. The analysis was performed in a single-blind manner by one person to avoid bias.

### Sperm Chromatin Structure Assay

A sperm chromatin structure assay was performed according to a previously described protocol (Evenson and Jost, 2001). This technique is based on the vulnerability of sperm DNA to acid-induced denaturation *in situ* and subsequent metachromatic staining with acridine orange. The DNA fragmentation index (DFI) lrb% and high DNA stainability (HDS) lrb%, indicators of chromatin immaturity (*i.e*., protamination completeness), were assessed. The samples were evaluated using a FACSVerse Flow Cytometer (BD Biosciences) controlled with BD FACSuite. For each sample, 5,000 events were recorded. Excitation of acridine orange was performed with a blue laser (488 nm); red fluorescence was detected with a 700/54 BP filter, and green fluorescence was detected with a 537/32 BP filter. Data were analyzed using WEASEL Ver. 3 (WEHI).

### Electrophoresis and Western Blot

Electrophoresis and Western blotting were performed on testicular tissue and the sperm of experimental males. Testes samples were lysed in radioimmunoprecipitation assay buffer enriched with a complete mini protease inhibitor cocktail (Roche, Switzerland). Samples of sperm heads were prepared by sonication and lysis in radioimmunoprecipitation assay buffer supplemented with 100 mM DTT. Protein concentration was measured using the bicinchoninic acid method (Lovrien and Matulis, 2005; Olson and Markwell, 2016). Thereafter, the samples were mixed with Laemmli loading buffer supplemented with β-mercaptoethanol. For dodecyl sulfate polyacrylamide gel electrophoresis, 4–15% separating Mini-PROTEAN^®^ TGX Stain-Free™ Precast Gels (Bio-Rad, France) were used; 30 μg of testicular proteins (60 μg) or sperm proteins were loaded into each gel chamber. For Western blotting, the Trans-Blot^®^ Turbo™ Transfer System (Bio-Rad, France) was used. Polyvinylidene difluoride membranes (Bio-Rad, France) were blocked in 5% bovine serum albumin in TBS with 0.5% Tween-20 for 60 min at 21 ± 1°C and incubated overnight at 4°C with primary antibodies diluted in 1% bovine serum albumin in TBS with 0.5% Tween-20. Molecular sperm quality was evaluated using a rabbit polyclonal anti-H3K4me2 antibody (1:1,000; cat. no. ab7766; Abcam, United Kingdom) and a mouse monoclonal anti-γH2AX antibody (1:1,000; cat. no. ab26350; Abcam, United Kingdom). Rabbit polyclonal anti-α-tubulin antibody (1:1,000; cat. no. #2144; Cell Signaling, United States) was used as the housekeeping antibody. Horseradish peroxidase-conjugated secondary antibodies (goat anti-mouse or anti-rabbit IgG; 1:15,000; Invitrogen, United States) were applied for 1 h at 21 ± 1°C. The targeted proteins were visualized using ECL Select Western blotting Detection Reagent (GE Healthcare Life Sciences, United Kingdom), and membranes were scanned on a ChemiDoc™ MP System (Bio-Rad, France). Images of membranes were processed using ImageLab 4.1 software (Bio-Rad, France). The same method was used to evaluate testicular tissue, which revealed changes in the context of spermatogenesis between experimental groups.

### Immunofluorescence

For γH2AX and H3K4me2 immunostaining, 10 sections were dewaxed, rehydrated, and processed as described by Chemek et al. (2018) with some modifications. Antigen retrieval was performed by pressure-cooking slides for 10 min in 0.01 M citrate buffer (pH 6.0). Non-specific binding sites were blocked with a solution of 5% normal goat serum (NGS) and 0.1% TritonX-100 and 0.5% Tween 20 in phosphate-buffered saline (PBS-TT) for 60 min at 21 ± 1°C. Subsequently, the testis sections were incubated with a rabbit polyclonal anti-H3K4me2 antibody (1:100; cat. no. ab7766; Abcam, United Kingdom) or a mouse monoclonal anti-γH2AX antibody (1:200; cat. no. ab26350; Abcam, United Kingdom) before overnight incubation at 4°C. Non-specific binding of the secondary antibodies was tested by omitting specific primary antibodies and these slides were processed at comparable settings. After washing in PBS-TT solution containing 1% NGS, slides were incubated for 40 min with PNA lectin (Alexa Fluor 488, Abcam, United Kingdom) diluted 1:400 and with the appropriate secondary antibody (anti-mouse or anti-rabbit Alexa Fluor 647, Abcam, United Kingdom) diluted 1:500 in PBS-TT containing 1% NGS. The slides were mounted with Vectashield medium plus 4,6-diamidino-2-phenylindole (DAPI; Vector Laboratories Inc., United States) for nuclear staining. Slides were then observed with a confocal microscope, and images were analyzed using ImageJ software (NIH, United States).

### Terminal Deoxynucleotidyl Transferase dUTP Nick End Labeling Assay

TUNEL assays were performed to analyze DNA double-strand breaks in blastocysts derived from zygotes flushed after exposed male mating. Fixed blastocysts were permeabilized in 0.1% Triton X-100 in PBS containing 0.05% NaN_3_ for 40 min. The blastocysts were then treated with fluorescein-conjugated dUTP and terminal deoxyribonucleotidyl transferase enzyme (*In Situ* Cell Death Detection Kit, cat. no. 11684795910, Roche, Germany) for 1 h in the dark at 37°C. The positive control was prepared using a DNase I kit (AMP-D1, Sigma-Aldrich, United States). Finally, the blastocysts were mounted onto slides with Vectashield medium and DAPI (Vector Laboratories Inc., United States). Signal intensity was measured using ImageJ software (National Institutes of Health, United States).

### Immunocytochemistry

Fixed zygotes were permeabilized in PBS containing 0.04% TritonX-100 and 0.3% Tween-20 for 15 min at 37°C. Zygote epigenetic marks were evaluated, including γH2AX and H3K4me2. Zygotes were then blocked in 1% bovine serum albumin in PBS with Tween-20 for 15 min and incubated with a cocktail of antibodies against mouse anti-γH2AX (1:200, cat. no. ab26350; Abcam, United Kingdom) and rabbit anti-H3K4me2 (1:200, cat. no. ab7766; Abcam, United Kingdom) antibodies for 1 h at 21 ± 1°C. Non-specific binding of the secondary antibodies was tested by omitting specific primary antibodies and these slides were processed at comparable settings. After washing, the oocytes were incubated with a cocktail of anti-mouse and anti-rabbit Alexa Fluor 488 and 647 (1:200) antibodies, respectively. Phalloidin (1:200; cat. no. #13054; Thermo Fisher Scientific, United States) was added to the wash and used for β-actin visualization. Stained zygotes were mounted onto slides in Vectashield medium with DAPI (Vector Laboratories Inc., Burlingame, CA, United States). Images were acquired using an Olympus IX83 spinning disc confocal microscope (Olympus, Germany) and VisiView software (Visitron Systems GmbH, Germany). The number of γH2AX loci in the developed paternal pronucleus was assessed. The integrated density of H3K4me2 in those pronuclei was measured using ImageJ (NIH, United States) software; the expanded pronucleus was considered as paternal, and the density was normalized to maternal density.

### Statistical Analyses

The data were analyzed using GraphPad Prism 8.1.1 (GraphPad Software Inc., San Diego, CA, United States). Based on Shapiro-Wilk’s normality distribution tests, differences were tested using an ordinary one-way analysis of variance, followed by Tukey’s multiple test. Alternatively, Kruskal-Wallis and Dunn’s *post hoc* tests were used for non-normally distributed data. *P*-values < 0.05, 0.01, 0.001, and 0.0001 were considered statistically significant and indicated with asterisks (*), (^**^), (^***^), and (^****^), respectively. Alternatively, daggers indicated differences from the positive control (low DES) (#, ##, ###, and ####, respectively). Normally and non-normally distributed data are expressed as means and medians, respectively.

## Results

### Effect of Nursing Exposure to Bisphenols on Reproductive Signs and the Spermiogram

**FIGURE 1:**
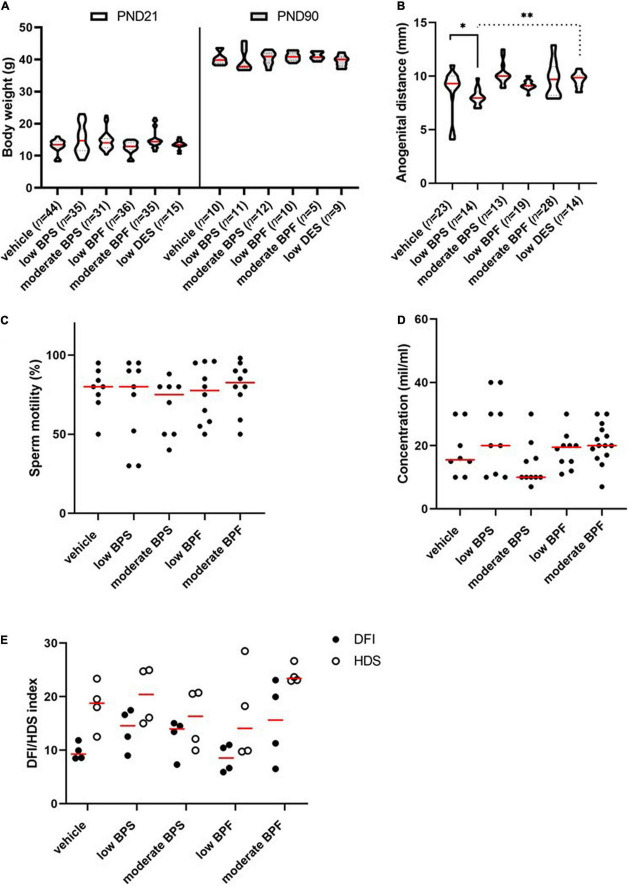
Morphological signs and spermiogram parameters of males exposed to bisphenols via maternal milk. **(A)** Comparison of weight (g) of male offspring in the post-natal day (PND) 21 and PND 90 in control, low bisphenol S (BPS), moderate BPS, low bisphenol F (BPF), moderate BPF, and low diethylstilbestrol (DES) groups. The shape of violin plots represents the distribution of weight in each experimental group, the red line shows median and dashed lines show quartiles. Numbers of analyzed offspring are indicated in brackets. **(B)** Anogenital distance (mm) of male offspring in control, low BPS, moderate BPS, low BPF, moderate BPF, and low DES groups. The shape of violin plots represents the distribution of anogenital distance in each experimental group, the red line shows median and dashed lines show quartiles. Numbers of analyzed offspring are indicated in brackets. **(C)** Motility of spermatozoa (%) of male offspring in control, low BPS, moderate BPS, low BPF, and moderate BPF groups. Dots represent individual males; red lines show the median. **(D)** Concentration of spermatozoa (mil/ml) on male offspring in control, low BPS, moderate BPS, low BPF, and moderate BPF groups. Dots represent individual males; red lines show the median. **(E)** DNA fragmentation index (DFI) of male offspring in control, low BPS, moderate BPS, low BPF, and moderate BPF groups and high DNA stainability index (HDS) of male offspring in control, low BPS, moderate BPS, low BPF, and moderate BPF groups. Dots represent individual males; red lines show the median. Statistical differences were tested using the Kruskal-Wallis test, followed by Dunn’s multiple comparisons test. Statistical differences are indicated from the vehicle (*P < 0.05, ^**^P < 0.01). For DFI and HDS, statistical differences were tested using the two-way ANOVA followed by Sidak’s multiple comparison test. BPF: bisphenol F; BPS: bisphenol S; PND: post-natal day; DES: diethylstilbestrol; DFI: DNA fragmentation index; HDS: high DNA stainability index.

In our experiment, male offspring were exposed to BPS or BPF during the first 15 days of their lives through breast milk. Diethylstilbestrol was used as an estrogen positive control. The aim was to elucidate bisphenols’ obesogenic and/or estrogen-like effect on morphological development of exposed males, due to body weight and anogenital distance, respectively. Moreover, the conventional sperm parameters and DNA integrity were assessed. There was no impact of exposure on the body weight of males at PND 21 or PND 90 (Figure 1A). However, low BPS exposure affects the anogenital distance, although the same dose of DES does not show any effect (Figure 1B). Sperm motility (Figure 1C) and sperm concentration (Figure 1D) were not significantly reduced in bisphenol-exposed groups; the DFI and sperm immaturity *via* HDS were also not affected (Figure 1E).

### Nursing Exposure to Bisphenols Changes Histone Code in Germ Cells of Adult Testis

**FIGURE 2:**
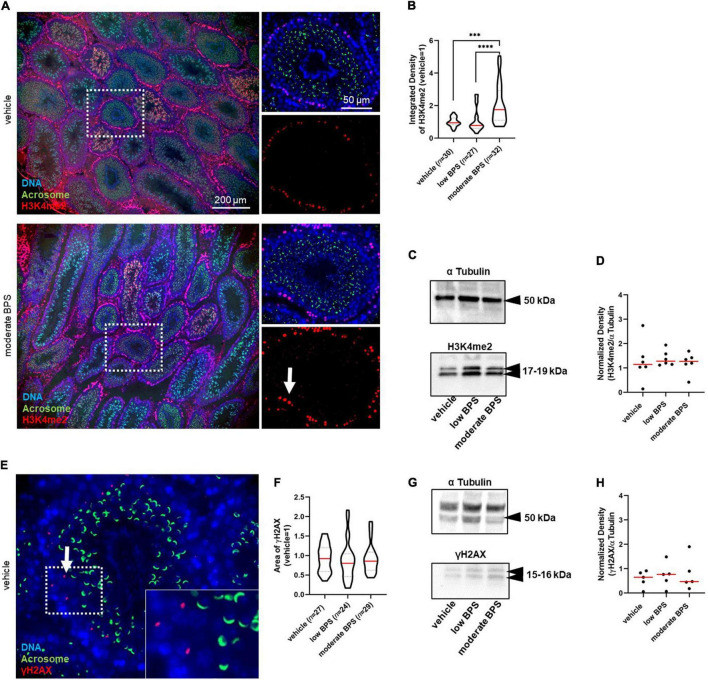
Epigenetic quality of germ cells in the testicular tissue of adult males exposed to bisphenol during the nursing period. **(A)** Representative microphotographs of dimethylation of lysine (K4) on histone H3 (H3K4me2) in seminiferous tubules (magnification 10 × and 60 ×). The arrow indicates H3K4me2 in the seminiferous tubule. **(B)** Quantification of the intensity of the H3K4me2 expression in seminiferous tubules at stage VII–VIII of spermatogenesis marked by red fluorescence in control, low bisphenol S (BPS), and moderate BPS groups. The shape of the violin plot represents the distribution in each experimental group, and red lines represent median and dashed lines show quartiles. Numbers of analyzed seminiferous tubules belonging to three individual males are indicated in brackets. **(C)** Dimethylated testicular H3 on lysine K4 (H3K4me2). **(D)** Density of H3K4me2 in testicular tissue; band signals were normalized to α tubulin. Dots represent individual males; red lines show the median. **(E)** Representative microphotographs of phosphorylation of histone H2 (γH2AX) expression in seminiferous tubules. The arrow indicates γH2AX in the seminiferous tubule. **(F)** Area of γH2AX expression in seminiferous tubules at stage VII–VIII of spermatogenesis in control, low BPS, and moderate BPS groups. The shape of the violin plot represents the distribution in each experimental group, red lines represent median, and dashed lines show quartiles. Numbers of analyzed seminiferous tubules belonging to three individual males are indicated in brackets. **(G)** Phosphorylated testicular H2AX (γH2AX). **(H)** Density of γH2AX in testicular tissue; band signals were normalized to α tubulin. Dots represent individual males; red lines show the median. The difference was tested using the Wilcoxon matched-pairs signed rank test. Asterisks indicate statistical differences (^***^P < 0.001, ^****^P < 0.0001). BPS: bisphenol S.

Because there was no observable effect of low doses of bisphenols as obesogenic, estrogen-like, and/or toxic compounds, we considered the epigenetic mode of bisphenol action toward early life-vulnerable germ cells. Therefore, we focused on well-established epigenetic markers of DNA damage—H3K4me2 and γH2AX. First, we evaluated the amount of H3K4me2 in seminiferous tubules at stages VII–VIII of spermatogenesis, as a parameter of DNA damage and integrity. These stages were recognized using FITC-conjugated peanut-agglutinin co-staining and corresponded to spermiation (Figure 2A). In the immunofluorescence assay, we observed a statistically significant increase in H3K4me2 levels in the testes of males exposed to moderate doses of BPS; however, there was no statistical difference between the control group and low BPS group (Figure 2B). We enhanced this finding with Western blotting and densitometry analysis, approved by the molecular weight of histone H3 (Figure 2C). In whole-testicular lysates, there was no statistical difference between the control and experimental groups (Figure 2D). In addition to H3K4me2, we analyzed the amount of γH2AX in seminiferous tubules at stages VII–VIII of spermatogenesis (Figure 2E). We analyzed the area of expression of the γH2AX and found no statistical difference between the control and experimental groups (Figure 2F). Neither Western blotting nor densitometry (Figure 2G) showed statistically significant differences between the control and experimental groups (Figures 2G,H).

### Bisphenol Nursing Exposure Does Not Affect Histone Code in Spermatozoa of Adult Males

**FIGURE 3:**
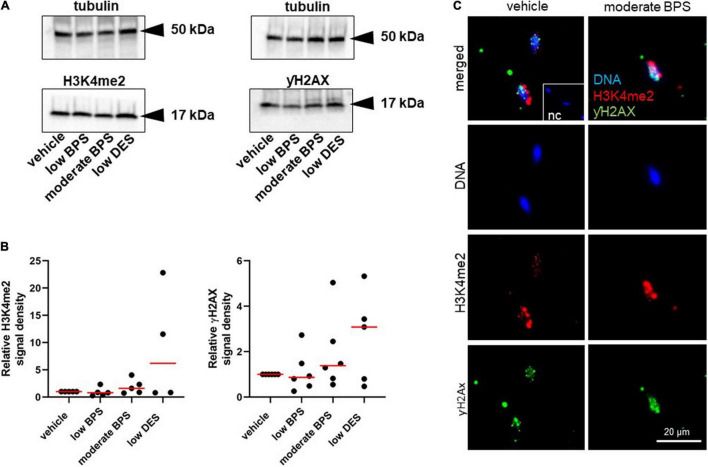
Epigenetic quality of spermatozoa of adult males exposed to bisphenol during the nursing period. **(A)** Representative Western blots of dimethylation of lysine (K4) on histone H3 (H3K4me2) and phosphorylation of histone H2 (γH2AX) of sperm-head lysates from control, low bisphenol S (BPS-), moderate BPS-, and low diethylstilbestrol (DES-) exposed males. **(B)** Densitometry analysis of H3K4me2 and γH2AX. Band signals were normalized to tubulin. Dots represent individual males, and red lines show the median. **(C)** Immunocytochemistry assay of H3K4me2 and γH2AX expression in mouse spermatozoa of vehicle control and moderate BPS; the NC was prepared by omitting a specific antibody and incubated with fluorescein-conjugated antibody. NC: negative control; BPS: bisphenol S; DES: diethylstilbestrol.

According to the results with testicular tissue, we determined the amount of DNA breaks due to the assessed epigenetic markers: the dimethylation of histone H3 (H3K4me2) and phosphorylation of histone H2 (γH2AX). Both markers were examined in spermatozoa by Western blotting, followed by quantification by densitometry (Figure 3A). There was no statistical difference between the control and experimental groups in the levels of H3K4me2 and γH2AX (Figure 3B). The specificity of antibody binding was elucidated by immunocytochemistry, and representative images of H3K4me2 and γH2AX localization in the sperm head are shown in Figure 3C.

### Nursing Exposure to Bisphenols Impairs Sperm Contribution on Zygote Quality

**FIGURE 4:**
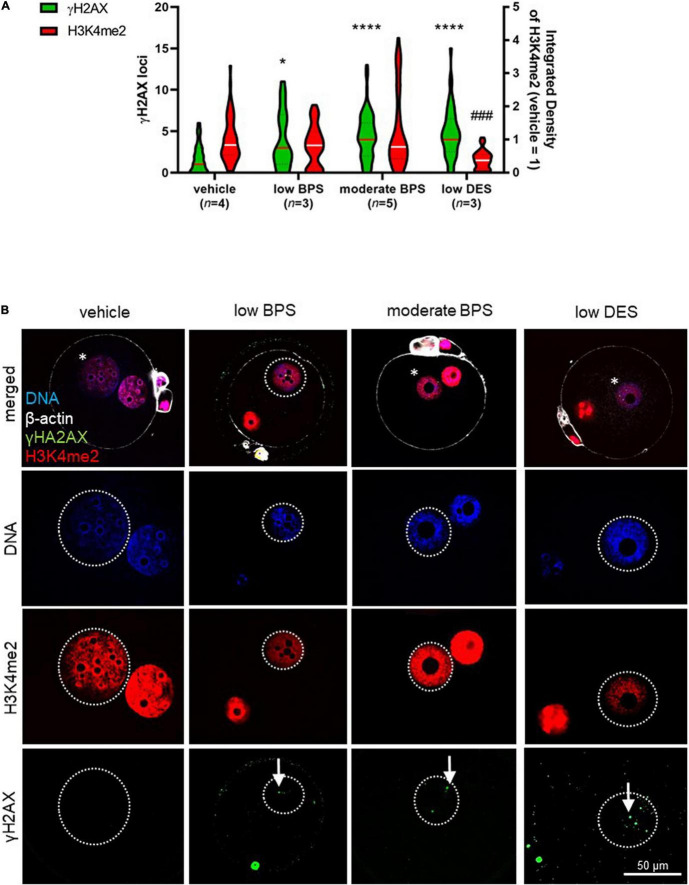
Zygote quality after fertilization by bisphenol-affected spermatozoa. **(A)** Number of phosphorylation of histone H2 (γH2AX) loci in paternal pronuclei, in low and moderate BPS and low DES groups. Relative signal intensity of dimethylation of lysine (K4) on histone H3 (H3K4me2) in paternal pronuclei in zygotes of control, low and moderate bisphenol S (BPS) and low diethylstilbestrol (DES) groups. Integrated density was normalized to maternal values of the respective zygote. Violin plot shows the distribution of individual values (numbers of independent flushing sessions are indicated in brackets), lines represent the median, and dashed lines show quartiles. **(B)** Representative pictures of DNA, H3K4me2, and γH2AX in zygotes. Comparison between experimental groups. Asterisks indicate paternal pronucleus. Arrows indicate γH2AX loci. Differences were tested using the Kruskal-Wallis test, followed by Dunn’s multiple comparisons test. Statistical differences in γH2AX loci and H3K4me2 integrated density are indicated by asterisks and daggers, respectively (*P < 0.05, ****P < 0.0001; ^###^*P* < 0.001). BPS: bisphenol S; DES: diethylstilbestrol.

According to the findings mentioned above, we assessed sperm chromatin quality after development to the paternal pronucleus, followed by an *in vivo* fertilization assay and zygote flushing. Zygotes were fixed, and H3K4me2 and γH2AX were co-stained using immunocytochemistry. Based on DNA staining of both pronuclei, progressive development was considered to be paternal. We analyzed the integrated density of H3K4me2 in the paternal pronuclei and observed decreased levels of H3K4me2 in the low DES group (Figure 4A), although no observable effect was observed in zygotes belonging to moderate-BPS-exposed males. In addition to H3K4me2, there was an increase in the number of γH2AX loci in all experimental groups compared to that in the vehicle control, indicating DNA damage in bisphenol-affected spermatozoa participating in fertilization. The exposure to low DES similarly induces increasing γH2AX (Figure 4A). Subcellular localization of both histone markers in the paternal pronucleus is shown in Figure 4B.

### Bisphenol Nursing Exposure Affects Sperm Development Competence

Following the findings of a higher occurrence of DNA damage markers in zygotes after BPS/BPF exposure, we evaluated early embryonic development up to the blastocyst stage. Therefore, *in vivo* produced zygotes were flushed and cultured *in vitro* until the blastocyst stage, alternatively to the zygote immunostaining mentioned above. In addition to this evaluation, we assessed the quality of blastocysts achieved *in vitro*, based on blastomere cell counting and DNA damage evaluation.

**FIGURE 5:**
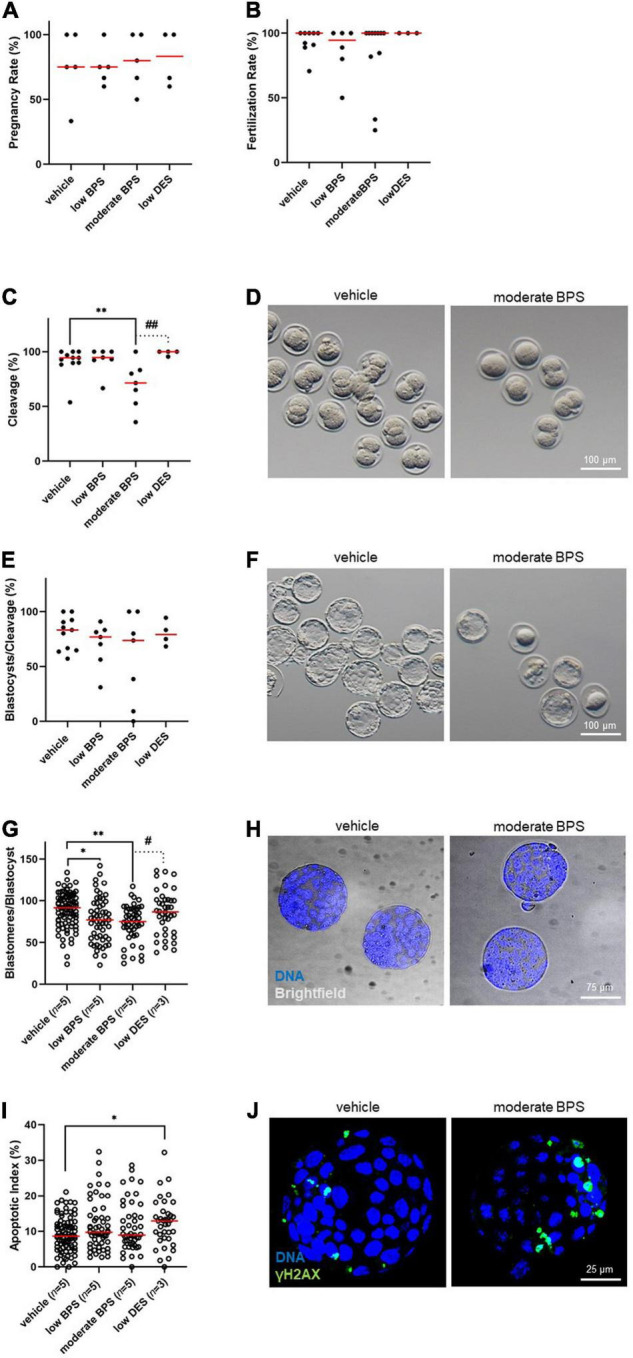
Development of the embryos is compromised by male bisphenol exposure during the nursing period. **(A)** Pregnancy rate (%) of females mated by bisphenol-exposed males of control, low bisphenol S (BPS), moderate BPS, and low diethylstilbestrol (DES) groups. Dots represent the values belonging to individual males, and the red line shows the median. **(B)** Fertilization rate (%) of males, expressed as a portion of fertilized oocytes, while females are recognized as pregnant and flushed. Dots represent values belonging to individual males, and the red line shows the median. **(C)** Cleavage of zygotes after BPS-exposed males (%) in the control, low BPS, moderate BPS, and low DES groups. Dots represent values belonging to individual males, and the red line shows the median. **(D)** Representative pictures of cleavage of zygotes on behalf of control and moderate-BPS-exposed males. **(E)** Blastocyst rate (%), expressed as the percentage of blastocysts related to cleaved embryos. Dots represent values belonging to individual males, and the red line shows the median. **(F)** Representative pictures of blastocysts of control and moderate BPS group. **(G)** Blastomeres per blastocyst in control, low BPS, medium BPS, and low DES groups. Dots represent values belonging to individual blastocysts acquired at least in three independent (*in vitro* culture) sessions, and the red line shows the median. Numbers of independent flushing sessions are indicated in brackets. **(H)** Representative pictures of control and moderate BPS blastocysts stained with 4,6-diamidino-2-phenylindole (DAPI). **(I)** Apoptotic index (%) in control, low BPS, moderate BPS, and low DES groups. Dots represent values belonging to individual blastocysts acquired at least in three independent (*in vitro* culture) sessions, and the red line shows the median. Numbers of independent flushing sessions are indicated in brackets. **(J)** Representative pictures of control and DES blastocysts, showing a difference in terminal deoxynucleotidyl transferase dUTP nick end labeling (TUNEL)-positive blastomeres. The difference was assessed using a one-way ANOVA, followed by Tukey’s multiple comparisons test. Statistical differences are indicated from the vehicle (*P < 0.05, ^**^P < 0.01) and positive control (low DES; ^#^*P* < 0.05, ^##^*P* < 0.01). ANOVA, analysis of variance; BPS, bisphenol S; DES, diethylstilbestrol, DAPI: 4,6-diamidino-2-phenylindole; TUNEL, terminal deoxynucleotidyl transferase dUTP nick end labeling.

First, we analyzed the pregnancy rate (Figure 5A) of females mated by bisphenol-exposed males, followed by the assessment of the fertilization rate as the ratio of zygotes to non-fertilized oocytes (Figure 5B). For both parameters, there was no statistical difference between the control and experimental groups. We then evaluated the quantitative indicators of embryonic development. For cleavage, there was a statistically significant decrease in the moderate BPS group by a median of 71.4% (Figures 5C,D). For the blastocyst rate, we did not record statistical differences between the control and moderate BPS groups when the blastocysts were related to cleaved embryos (Figures 5E,F). Although the amount of blastocysts dropped by 41.2% in the moderate BPS group (data not shown), we considered this to be a result of embryo cleavage. Qualitative indicators of embryonic development, such as the number of blastomeres per blastocyst and apoptotic index, were evaluated using DAPI staining and TUNEL assay. There was a statistical difference between the control group and both BPS groups in the number of blastomeres per blastocyst (Figures 5G,H). There was a statistical difference in the apoptotic index between the control and low DES groups (Figure 5I), while DES-derived blastocysts showed surprisingly increased blastomere counts. Obviously, zygotes derived from BPS-exposed males are affected in terms of further embryonic development. Similarly, the quality of blastocysts is affected in BPS-exposed males, although the incidence of DNA-damaged blastomeres does not increase. We also discovered an increase in γH2AX in blastomeres in the moderate BPS group by the TUNEL assay (Figure 5J).

### Embryo Success Is Determined by Sperm Quality Achieved During Spermatogenesis

We used data from previous experiments to determine the statistical correlations between individual parameters. We used the Spearman correlation coefficient to evaluate dependencies, which we supplemented with confidence intervals. The expected relationship between the fertilization rate and blastocyst rate was confirmed based on the finding of a moderate positive correlation. Interestingly, we found a moderate negative correlation between H3K4me2 in testicular germ cells and cleavage of early embryos, as well as a high negative correlation between H3K4me2 and double-strand breaks in blastocysts. Although no difference in the phosphorylation of sperm histone H2AX (γH2AX) was observed between control and bisphenol-exposed adult males, there is a high positive correlation between γH2AX in spermatozoa and γH2AX loci in paternal pronuclei in zygotes. It is clear that testicular quality and sperm quality are associated with the success of early embryonic development and blastocyst quality, as well as the vulnerability of juvenile testis to environmental noxious influences. All significant relations are summarized in Table 1. For complete outputs of Spearman coefficients and confidence intervals, see Supplementary Table 1.

**TABLE 1:** Correlations between selected parameters.

	Anogenital distance	Motility	Concentration	Testes H3K4me2	Sperm H3K4me2	PN γH2AX	Pregnancy rate	Cleavage	Blastocyst amount	Blastocyst rate	Blastomeres per blastocyst	TUNEL
Treatment				0,738					−0,498 −0,7954 to −0,007216	−0,489 −0,7912 to 0,003957		
W dam (PND 10)			−0,423 −0,7175 to −9,525e−006				−0,660 −0,8698 to −0,2485					
W% gain (PND 10)	−0,372 −0,6333 to −0,03352		−0,614 −0,8308 to −0,2350			0,687 0,2010 to 0,9015						
Average body weight (PND 21)	*0,527* 0,2646 to 0,7170											
Body weight (PND 90)		−0,550 −0,8204 to −0,07913										
Motility												−0,886
Concentration					0,746 0,3834 to 0,9093							
Testes H3K4me2								−0,650				−0,733
Sperm H2AX						0,663						
Pregnancy rate											−0,512 −0,7836 to −0,07574	
Fertilization rate									0,651			
Cleavage												0,498
Blastocyst rate (per total)										*0,874* 0,6968 to 0,9508		

*Spearman coefficients are supplemented by confidence intervals. PND: post-natal day; TUNEL: terminal deoxynucleotidyl transferase dUTP nick end labeling. Values of Spearman correlation coefficient are signifficant at P ≤ 0.05, italicized values are at P ≤ 0.01.*

## Discussion

Bisphenols are widespread endocrine disruptors, and their effects on reproductive health are currently intensively disputed. Our study contributes to this issue by providing a unique method of exposure *via* breastfeeding. Although there is no evidence that the concentration of bisphenols is higher in human breast milk than in adult human urine or serum (Vandenberg et al., 2010; Dualde et al., 2019; Luo et al., 2021), nursing exposure of infants is considered dangerous due to the exclusiveness of breast milk as the source of nourishment. Moreover, newborns do not have fully developed elimination mechanisms involving detoxification in the liver and/or the glucuronidation pathway in the kidney (Matalová et al., 2016). Although the chosen dosage was extremely low in accordance with the actual exposure (González et al., 2020; Gys et al., 2020; Kim et al., 2020; Luo et al., 2021), we expected higher intake per gram of body weight of infants, leading to significant damage to germ cells and fertility while achieving adulthood, as indicated earlier in our previous findings (Nevoral et al., 2021).

We determined that nursing exposure to BPS and BPF did not affect fundamental spermiogram parameters such as motility, concentration, DFI, and HDS of spermatozoa of male mice exposed via breast milk, which is in accordance with a previous observation (Ullah et al., 2019); although direct BPS exposure of adult male mice through drinking water decreased the portion of motile spermatozoa (Řimnáčová et al., 2020). Therefore, we can assume that sperm motility affects the route of exposure and/or maturity of the exposed organism. Apparently, individual bisphenols can show different modes of action (Shi et al., 2017; Ullah et al., 2018) and, consequently, bisphenol substitutions (Eladak et al., 2015) do not seem to be a reliable approach for the elimination of endocrine disrupting effects (Žalmanová et al., 2016). We presumed that the doses of bisphenols used in our experiments influenced spermatogenesis in a more delicate way, such as through epigenetics, without any observable phenotype in terms of motility and/or sperm concentration.

Male mice were exposed to alternative bisphenols, BPS and BPF, through maternal milk in the neonatal exposure window, when the differentiation of germ cells and dynamic epigenetic changes take place (Ernst et al., 2011; Nakata et al., 2015). Hence, we used epigenetic assessment of testicular tissue by H3K4me2 and γH2AX. The H3K4me2 is a well-known marker of DNA damage in many types of body tissues (Zhang et al., 2012; Wang et al., 2020). In spermatozoa, H3K4me2 serves as a marker of immaturity and indicates aberrant histone-protamine exchange, resulting in improper chromatin decondensation (Rahman et al., 2017). Katz et al. (2009) discovered that accumulation of H3K4me2 in testicular tissue leads to sterility. Indeed, exposure to BPS increased the levels of H3K4me2 in spermatogonia and spermatocytes as shown via *in situ* immunofluorescence but not Western blot densitometry, supporting the relevance of middle stage selection for assessment. Similarly, γH2AX is a stable marker of DNA double-strand breaks and DNA integrity (Derijck et al., 2006; Kuo and Yang, 2008; Sharma et al., 2012). To the best of our knowledge, we tracked γH2AX and analyzed seminiferous tubules at the middle (VII-VIII) stages of spermatogenesis, which correspond with rendering round spermatids in the adluminal compartment (Nakata et al., 2015); round spermatids are beyond the physiological pachytene DNA breaks occurring in crossing over. Interestingly, γH2AX expression did not show any difference in testicular tissue, even in specified seminiferous tubules at stages VI-VIII. We assumed a bisphenol-modulated epigenetic shift in primary and secondary spermatocytes in seminiferous tubules, which can denote a burden transmitted to spermatozoa of adult males (Ventelä et al., 2002). We did not observe any epigenetic phenotype using the lysate of matured sperm, indicating that epigenetic damage can be repaired during further spermatogenesis. Indeed, we did not observe any correlation between H3K4me2 in testicular tissue, sperm lysate, or H3K4me2 in the male pronucleus. Neither testicular nor sperm H3K4me2 showed a relationship with blastocyst rate, which indicates sperm restoration without any impact of this sperm-born epigenetic parameter. Apparently, the method of exposure plays a crucial role, while spermatogonia are affected as possible remain of earlier damaged germ cells.

For more rigorous testing of sperm fitness, we further assessed the density of the sperm head after physiological decondensation under oocyte conditions. Accordingly, nursing-exposed males failed to show early embryonic development and showed decreased blastocyst rates in flushed zygotes. Although the impact of damaged spermatozoa on early embryonic development has been previously described (Sedó et al., 2017; Rajabi et al., 2018; Wyck et al., 2018; Casanovas et al., 2019; Middelkamp et al., 2020), our study highlights the features of early life-born sperm. Although this sperm molecular signature seems to be subtle and minor in relation to the manifestation, it is obviously responsible for the ability of sperm fertilization, in addition to other features, such as motility and/or concentration.

According to our observations, γH2AX is transferred from the spermatozoon to the zygote and then to the blastocyst; thus, we assume that corruption is transmitted, even from germ cells, through spermatogonia into the spermatid and spermatozoon (Olsen et al., 2005). DNA double-strand breaks are physiologically generated during the onset of the prophase of meiosis I to allow crossing, followed by a DNA damage response due to homologous recombination (Meng et al., 2019). Double-strand breaks persist until round spermatids if DNA damage repair is impaired (Cordelli et al., 2012). DNA damage also occurs during protamination. Godman et al. (Godmann et al., 2007) reported that elongating spermatids increases H3K4me2. After protamination, there is the last chance for spermatozoa to repair DNA damage, and H3K4me2 becomes a relevant marker of inappropriate changes in these stages of spermiation. Reparations are terminated as transcription and translation stop post-spermiogenesis; thus, spermatozoa are unable to repair further DNA damage acquired during transit through the epididymis and post-ejaculation. Paternal DNA repair is again activated after fertilization, due to the oocyte repair mechanisms. The effect of spermatozoa DNA fragmentation on early embryonic development depends on the combination of the magnitude of these DNA defects and the capacity of oocytes for reparations (González-Marín et al., 2012). In our experiment, bisphenol treatment did not show any significant effect on the presence of γH2AX in spermatozoa; however, the impact of early life-borne DNA damage of germ cells on sperm and embryo quality is noteworthy. In addition, γH2AX is transmitted from spermatogonia to spermatozoa, and our observations showed that the individual γH2AX pattern in sperm cells corresponds with γH2AX loci in paternal pronuclei and further embryonic development. In addition, H3K4me2 is transferred from the spermatozoa to the paternal pronuclei. Our findings are supported by recent studies that have shown the impact of damaged spermatozoa on early embryonic development (Sedó et al., 2017; Rajabi et al., 2018; Wyck et al., 2018; Casanovas et al., 2019; Middelkamp et al., 2020).

The first mitotic division occurs along with the fusion of the paternal and maternal pronuclei. All proteins needed for that event have to be synthesized in oocytes before fertilization, except centrioles, which are brought into the zygote by spermatozoa (Sutovsky and Schatten, 2000). Following sperm acrosomal exocytosis, penetration of the *Zona pellucida*, and oocyte cortical reaction, the first mitotic division is a crucial checkpoint for further embryonic development (Stitzel and Seydoux, 2007; Kim et al., 2008). In our study, we observed the impact of bisphenol-affected male gametes on this first mitotic division (Tesarik, 2005; Derijck et al., 2008; Gawecka et al., 2013), and moderate doses of bisphenol impaired DNA integrity in the paternal pronucleus. Accordingly, the cleavage rate was significantly decreased by 71.4% compared to that in the control group.

In a mouse two-cell embryo, the embryonic genome is activated and becomes transcriptionally active (Svoboda, 2018). This is another checkpoint for early embryonic development, and the quality of the genome of the developing embryos is crucial. Because the decreased blastocyst rate followed after cleavage was impaired, we can assume that embryonic genome activation was not affected by bisphenol. This assumption is supported by the fact that the fertilization rate is positively correlated with the blastocyst rate. Moreover, blastocysts from both low and moderate BPS groups contained fewer blastomeres than blastocysts in the control group. We also observed an increase in DNA double-strand breaks marked by TUNEL-positive blastomeres belonging to the moderate BPS group. Alongside these findings, we discovered an important correlation between γH2AX in spermatozoa and γH2AX in the paternal pronuclei. We can thus assume that γH2AX in spermatozoa is a relevant marker of zygote quality and embryonic development success (Derijck et al., 2006; Turinetto et al., 2012; Wyck et al., 2018).

To the best of our knowledge, our study provides the first evidence of the biological consequences of germ cell-borne DNA damage on embryo quality. These findings provide impactful knowledge for human reproduction and/or sperm selection for assisted reproductive therapies, challenges related to idiopathic infertility, and the failure of *in vitro* production of human embryos. We can assume that γH2AX and H3K4me2 are important indicators of sperm quality and thus, embryonic quality, because of significant correlations through whole spermatogenesis and early embryonic development.

In conclusion, this study confirms that male exposure to endocrine disruptors, such as bisphenols, during the perinatal period through maternal milk affects the quality of germ cells and thus spermatozoa, zygotes, and early embryonic development. This finding can be the answer to idiopathic infertility and the postponed burden of infertility achieved in early life.

## Data Availability Statement

The original contributions presented in the study are included in the article/Supplementary Material, further inquiries can be directed to the corresponding author/s.

## Ethics Statement

The animal study was reviewed and approved by Animal Welfare Committee of the Ministry of Education, Youth and Sports of the Czech Republic, approval ID MSMT-11925/2016-3.

## Author Contributions

JN and MK: project conception. JN and PK: animal experimental design. TF, HŘ, MC, JH, and JN: execution of experiments. TF, HŘ, MC, and JN: compiling the results. JN: statistics and proofreading. TF and JN: writing the manuscript and data interpretation. All authors read and approved the final manuscript.

## Conflict of Interest

The authors declare that the research was conducted in the absence of any commercial or financial relationships that could be construed as a potential conflict of interest.

## Publisher’s Note

All claims expressed in this article are solely those of the authors and do not necessarily represent those of their affiliated organizations, or those of the publisher, the editors and the reviewers. Any product that may be evaluated in this article, or claim that may be made by its manufacturer, is not guaranteed or endorsed by the publisher.

## Abbreviations

BPs: bisphenols
BPA: bisphenol A
BPS: bisphenol S
BPF: bisphenol F
DAPI: 4,6-diamidino-2-phenylindole
DFI: DNA fragmentation index (DFI)
HDS: high DNA stainability
H3K4me2: dimethylation of lysine (K4) on histone H3
PBS: Phosphate buffered saline
PND: post-natal day
TUNEL: terminal deoxynucleotidyl transferase dUTP nick end labeling
γ H2AX: phosphorylation of histone H2.
